# Supplementary material for: Emulated retinal image capture (ERICA) to test, train and validate processing of retinal images
Source: Sci Rep. 2021 May 27;11:11225. doi: 10.1038/s41598-021-90389-y (PMC8160341; doi:10.1038/s41598-021-90389-y)
Supplement: Supplementary file 1 — Supplementary Information [file 41598_2021_90389_MOESM1_ESM.pdf]

Emulated Retinal Image CApture (ERICA) to test, train and validate processing of retinal images

Laura K Young[1,*] **and Hannah E Smithson**[2]

[1]Biosciences Institute, Newcastle University, Newcastle, NE2 4HH, UK
[2]Department of Experimental Psychology, University of Oxford, Oxford, OX2 6GG, UK
[*]laura.k.young@newcastle.ac.uk

Supplementary materials

Supplementary Videos 1 (noise and residual aberration), 2 (noise and diffraction-limited resolution) and 3 (diffraction-limited resolution) show a sequence of 90 synthetic frames at a retinal eccentricity of 3° generated using ERICA that constitute a 3-second movie. The scale bar represents 10 arcminutes. Encoded in the frames are noise and variations in image quality due to residual aberrations not corrected by the optical system, which are modeled using a a dataset of wavefront measurements captured from real eyes[1]. The synthetic frames have been generated with modeled movements of the eye , which are given in Figure 3. To generate these frames we simulated our AOSLO which has the properties shown in Table 1.

Parameter	value	units
Fast scan frequency	16	kHz
Slow scan frequency	30	Hz
Pinhole diameter	2	Airy radii
Number of pixels per line	320	pixels
Number of lines excluding flyback	525	lines
Number of lines including flyback	515	lines
Pixel size	0.22	arcmin
Imaging wavelength	850	nm
Pupil diameter	5	mm
Noise (mean)	0.35	gray level (0-1)
Noise (standard deviation)	0.07	gray level (0-1)

Table 1. The parameters that were used to generate Supplementary Videos 1 (noise and residual aberration), 2 (noise and diffraction-limited resolution) and 3 (diffraction-limited resolution), matching our AOSLO[2].

References

1. Jarosz, J. *et al.* High temporal resolution aberrometry in a 50-eye population and implications for adaptive optics error budget. *Biomed. Opt. Express* **8**, 2088, DOI: 10.1364/BOE.8.002088 (2017).

2. Young, L. K., Morris, T. J., Saunter, C. D. & Smithson, H. E. Compact, modular and in-plane AOSLO for high-resolution retinal imaging. *Biomed. Opt. Express* **9**, 4275, DOI: 10.1364/BOE.9.004275 (2018).
